# Proresolution Lipid Mediators in Multiple Sclerosis — Differential, Disease Severity-Dependent Synthesis — A Clinical Pilot Trial

**DOI:** 10.1371/journal.pone.0055859

**Published:** 2013-02-08

**Authors:** Harald Prüss, Berit Rosche, Aaron B. Sullivan, Benedikt Brommer, Oliver Wengert, Karsten Gronert, Jan M. Schwab

**Affiliations:** 1 Department of Neurology & Experimental Neurology, Charité University Medicine Berlin, Berlin, Germany; 2 Vision Science Program, School of Optometry, University of California, Berkeley, California, United States of America; Fundação Oswaldo Cruz, Brazil

## Abstract

**Background:**

The severity and longevity of inflammation is controlled by endogenous counter-regulatory signals. Among them are long-chain polyunsaturated fatty acid (PUFA)-derived lipid mediators, which promote the resolution of inflammation, an active process for returning to tissue homeostasis.

**Objective:**

To determine whether endogenous production of lipid-derived resolution agonists is regulated differentially in patients with highly active and less active multiple sclerosis (MS).

**Design:**

Matched-pairs study in University hospital Neurology department.

**Patients:**

Based on clinical (relapse frequency) and paraclinical (MRI lesions, contrast enhancement) criteria, 10 pairs of age- and sex-matched patients with relapsing-remitting MS were assigned either to a group with highly active or less active MS. Lipid mediators were quantified in serum and cerebrospinal fluid using LC-MS/MS-based lipidomics.

**Results:**

Levels of the key arachidonic (ω-6) and docosahexaenoic acid (ω-6)-derived mediators prostaglandins (PG), leukotrienes, hydroxyeicosatetraenoic acids (HETE) and resolution agonists lipoxin A_4_ (LXA_4_), resolvin D1 (RvD1) and neuroprotectin D1 (NPD1) were quantified. In the patient group with highly active MS, 15-HETE and PGE_2_ were increased, which are products of the 15-lipoxygenase and cyclooxygenase pathways. The proresolution mediator RvD1 was significantly upregulated and NPD1 was detected in the highly active group only. LXA_4_ levels were not increased in patients with highly active MS.

**Conclusions:**

Lipid mediator pathways are regulated differentially in the cerebrospinal fluid of MS patients, depending on disease severity. Non-exhaustive or possibly ‘delayed’ resolution pathways may suggest a defective resolution program in patients with highly active MS. Longitudinal analyses are required to hetero-typify this differential resolution capacity, which may be associated with disease progression, longevity and eventual termination.

## Introduction

Resolution of inflammation is an active endogenous process necessary for the termination of inflammatory and autoimmune diseases – inflammation does not just passively fizzle out. [1] It is a tightly controlled, directed tissue response orchestrated by cellular and humoral factors such as PUFA (omega- 3 (ω-3), -6 (ω-6))-derived lipid mediators. [1]–[3] Early interventional MS trials supplementing ω-3 and ω-6 were based on socio-epidemiological studies demonstrating that the risk of MS is high in countries with a high intake of saturated fatty acids and low in countries with a high intake of poly-unsaturated fatty acids (PUFAs). [4] This however remains controversial, since some ω-3 and -6-derived lipid mediators have revealed anti-inflammatory and others pro-inflammatory functions. [1].

It is noteworthy that arachidonic acid (AA)-derived prostaglandins such as prostaglandin E_2_ (PGE_2_) were originally identified primarily for their potent pro-inflammatory effect, promoting edema formation and oxidative toxicity, but they also promote T-helper (TH)1 and TH17 cell generation, which mediate tissue damage and inflammation. [5] Interference with its receptor suppresses disease progression in mice subjected to experimental autoimmune encephalomyelitis (EAE). [5] Elevated levels of PGE_2_ were detected in EAE lesions and in the cerebrospinal fluid (CSF) of MS patients. [6] On the other hand, AA is also a substrate for the bifunctional, anti-inflammatory and pro-resolutive molecule LXA_4_. [7], [8] The discovery of ω-3 and ω-6 as substrates for resolution agonists such as lipoxins, protectins and resolvins, which are generated by distinct transcellular pathways, provided a consistent rationale for the underlying biological functions. [1] Deficiencies in these resolution pathways can prolong inflammation and lead to the failure of tissue to return to homeostasis. [1] The recent identification of resolution agonists supports the notion that resolution is an active process independent of inflammation. [2].

However, the role of these molecules in MS pathology remains elusive. We therefore used LC-MS mass spectrometry to investigate the synthesis of the ω-3 and ω-6-derived resolution agonists in CSF and in serum samples of MS patients with highly and less active disease.

## Methods

### Patients

20 selected patients with a diagnosis of relapsing-remitting MS or clinically isolated syndrome according to the revised McDonald criteria from 2011 [9] were age- and sex-matched for highly or less active disease severity and enrolled in this cohort study. The trial considered samples from two treating Charité Neurology Centers (Campus Mitte and Campus Virchow). CSF and serum specimens were collected, immediately put on ice and a separate tube snap-frozen and stored in liquid nitrogen, all sample transportation was done on dry ice. According to the German Multiple Sclerosis Consensus Group [MSTKG] severe disease is considered with ≥6 lesions in the initial MRI or a severe clinical presentation at disease onset defined by an Expanded Disability Status Scale (EDSS) of ≥3 (because patients with a clinically isolated syndrome and EDSS ≥3 had a high probability to develop definite MS). Based on these criteria we scored all patients at initial diagnosis for (i) T2-weighted lesions in MRI (0–6 lesions = 1; 7–10 lesions = 2; >10 lesions = 3), (ii) gadolinium enhancing lesions (0 lesions = 1, 1 lesion = 2, >1 lesions = 3), (iii) EDSS (EDSS 0 = 1; EDSS 1–2 = 2; EDSS >2 = 3) and (iv) CSF cell count (normal [<15/3 per µl] = 1, ≤20/3 = 2; >20/3 = 3). Patients with a sum score ≤6 were classified as having less active MS and patients with >6 points as highly active MS. All patients had CSF oligoclonal bands. At time of investigation no patient had concurrent illnesses, was on treatment with steroids, immunosuppressive substances or was receiving diet supplements with long-chain PUFA. The Charité University Hospital ethical committee approved the experiments and all patients gave informed consent for research and publication.

### Lipid Mediator Analysis

**Figure 1:**
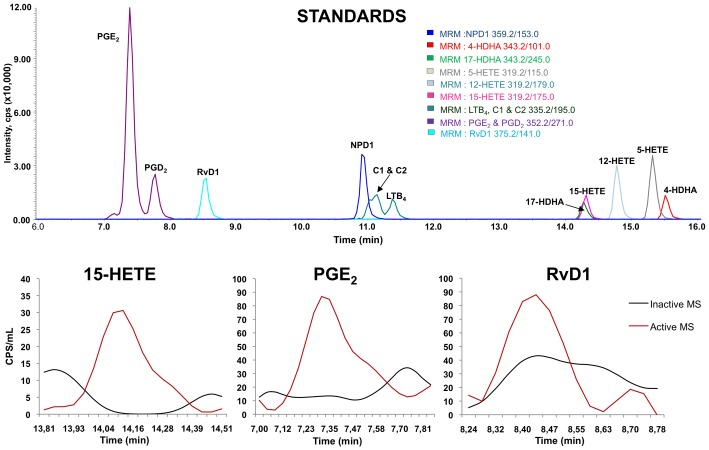
LC/MS/MS Lipid Mediator Profiling. A) MRM profile of 12 eicosanoid and docosanoid synthetic standards (NPD1, 4-HDHA, 17-HDHA, RVD1, PGE_2_, PGD_2_, LTB_4_, 6-trans LTB_4_ (C1), 6-trans-12-epi-LTB_4_ (C2), 12-HETE, 15-HETE, 5-HETE). B) Representative MRM chromatogram of 15-HETE, PGE_2_ and RvD1 from patients with less active MS (black line) and with highly active MS (red line). MRM signals (CPS) were corrected for recovery and cerebrospinal fluid sample size. Calibration curves (1–1000 pg) and specific LC retention times for each compound were established with synthetic standards (Cayman Chemical, Ann Arbor, MI). Structures were confirmed for selected samples by MS/MS analyses using enhanced product ion mode with appropriate selection of the parent ion in quadrupole 1.

For endogenous lipid mediator analysis, blinded snap frozen serum and CSF samples were rapidly thawed and immediately combined with two volumes of cold methanol (4°C) containing deuterated internal standards prostaglandin E_2_ (PGE_2_-d_4_), 15(S)-hydroxyeicosatetraenoic acid (15(*S*)-HETE-d_8_) and (leukotriene B_4_ LTB_4-_d_4_), arachidonic acid (AA-d8) and docosahexaenoic acid (DHA-d5) (400 pg/each) to calculate the recovery of different classes of oxygenated polyunsaturated fatty acids and their substrates (AA, DHA) (figure 1). Lipid autacoids were extracted by solid phase using SampliQ ODS-C18 cartridges (Agilent Technologies, Santa Clara, CA). Endogenous levels of the following lipid mediators were identified and quantified with LC/MS/MS-based lipidomics (figure 1): 4-, 7-, 14- and 17-hydroxy-docosahexaenoic acid (4-HDHA, 7-HDHA, 14-HDHA, 17-HDHA), 5-, 12- and 15-hydroxyeicosatetraenoic acid (5-HETE, 12-HETE, 15-HETE), prostaglandin E_2_, D_2_ and F_2_ (PGE_2_, PGD_2_, PGF_2_), lipoxin A_4_ and B_4_ (LXA_4_, LXB_4_), leukotriene B_4_ (LTB_4_), thromboxane B_2_ (TXB_2_), arachidonic acid (AA), docosahexaenoic acid (DHA), eicosapentanoic acid (EPA), resolvin D1 (RvD1), and neuroprotectin D1 (NPD1). [10]–[14] In brief, we analyzed extracted samples by a triple-quadrupole linear ion trap LC/MS/MS system (MDS SCIEX 3200 QTRAP) equipped with a Kinetex C18 mini-bore column. The mobile phase was a gradient of A [water/acetonitrile/acetic acid (72:28:0.01, v:v:v)] and B [isopropanol/acetonitrile (60:40, v:v)] with a 450 µl/min flow rate. MS/MS analyses were carried out in negative ion mode and prominent fatty acid metabolites were quantified by multiple reaction monitoring (MRM mode) using established transitions (table S1 and www.lipidmaps.org). [10] Average recovery rates for internal standards was monohydroxy PUFA (15-HETE) 48±3%, dihydroxy PUFA (LTB_4_/a 5,12-diHETE) 38±2%, trihydroxy PUFA (LXA_4_, a 5,6,15 triHETE) 39±2%, prostaglandins (PGE_2_) 54±2%, AA 21±1% and DHA 15±1%. Linear calibration curves (0.5–1000 pg or 0.01–10 ng for PUFA) and specific LC retention times for each analyte were established with synthetic standards (Cayman Chemical, Ann Arbor, MI). Limits of quantification for specific analytes ranged from 1–50 pg with a signal to noise ratio of >10. Structures were confirmed for selected autacoids by MS/MS analyses using enhanced product ion mode with appropriate selection of the parent ion in quadrupole 1.

### Statistical Analysis

The serum and CSF levels of lipid mediators (mean ± SEM) were compared between the two groups. Wilcoxon matched pairs test of significance (two-tailed) was performed with *P*<0.05 being considered significant.

## Results

A total of 20 subjects were enrolled in a matched paired cohort study. The age (mean ± SEM) in the ‘less active’ MS group (31.4 years ±2.5) was not statistically different from the ‘highly active’ group (34.0±2.3). Cohorts were composed of 5 male plus 5 female subjects each. Patients with highly active MS revealed higher (p = 0.0019; Mann-Whitney, two-tailed) numbers of CSF cells (40.2/3±8.6/3 per µl) compared to the less active group (9.4/3±1.97/3 per µl). Furthermore, the number of MS lesions in MRI was about three times higher (p = 0.0015) in the ‘highly active’ group (12.8±3.8) than in the ‘less active’ group (3.4±0.67).

**Figure 2:**
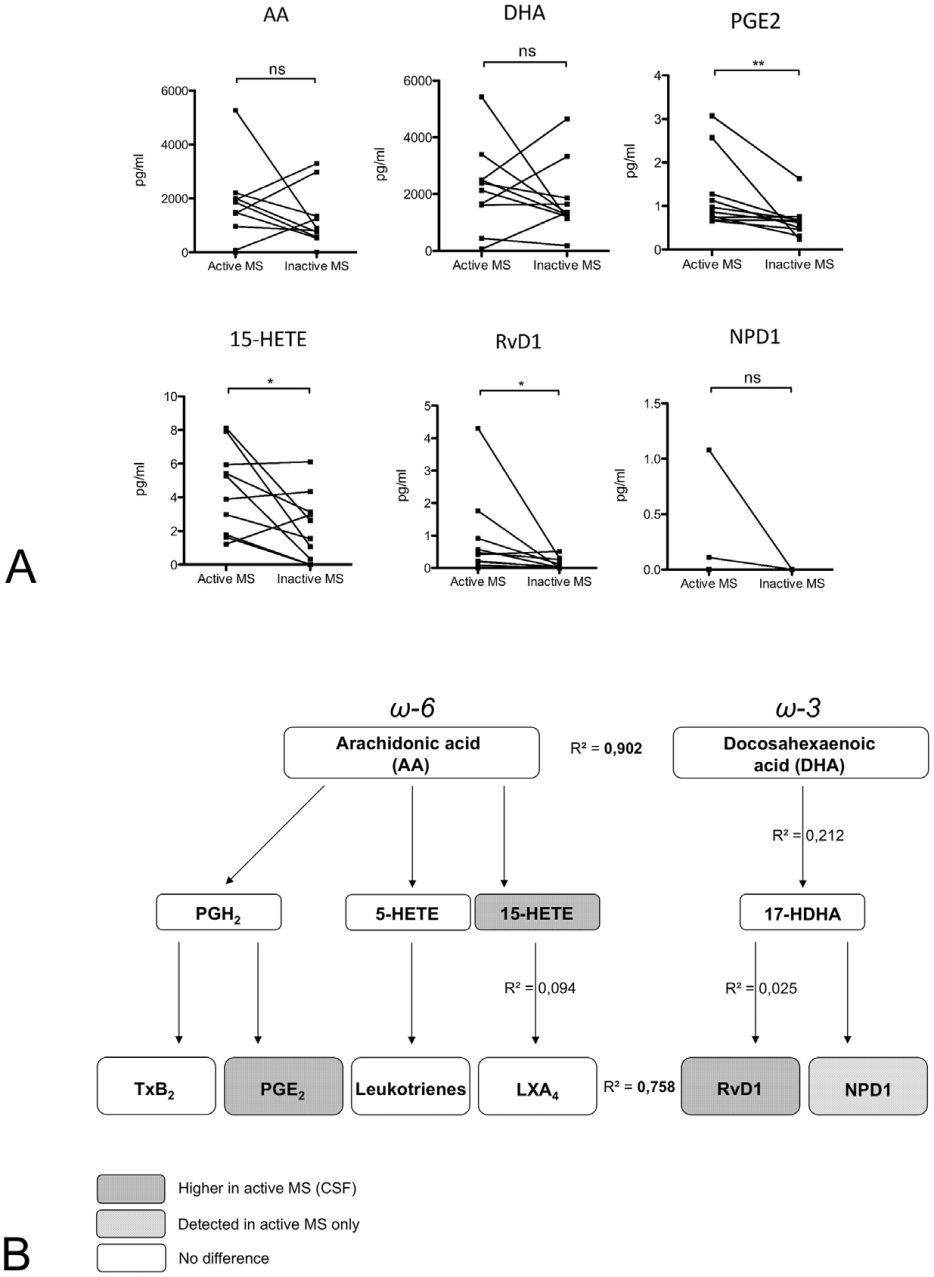
Pro-inflammatory and pro-resolution lipid mediators in CSF of patients with ‘highly active’ versus ‘less active’ MS. Released AA is converted into prostaglandins, leukotrienes, thromboxane, lipoxins, and hydroxy-eicosatetraenoic acids (HETEs), collectively termed eicosanoids. Lipoxygenase metabolism of DHA results in the generation of 17-HDHA, the resolvin D series and neuroprotectin D1. (A) Mass spectrometry detection in lumbar CSF suggests that the differences in the brain are very robust as the molecules are produced locally and become diluted during CSF flow. Precursors AA and DHA were similarly present in both disease phenotypes. PGE_2_, 15-HETE and the proresolution agonist RvD1 were significantly increased in patients with highly active MS, and NPD1 was only detectable in this group. (B) Synopsis of lipid mediator synthesis pathways and disease severity-dependent differences in CSF of MS patients. The levels of the precursors AA and DHA correlated closely, suggesting parallel regulation of the lipid mediator metabolism, but they did not correlate with active MS. In contrast, increased CSF levels of PGE_2_, 15-HETE and RvD1 correlated with active MS. Thus, lipid mediator production in active MS is not regulated by the release of precursor molecules but rather likely by the activation/expression of targeted enzymatic oxidation pathways as a specific disease response or component of disease progression.

Mass spectrometry analysis of serum and CSF samples was performed blinded and revealed significant differences in lipid mediator levels of both AA and DHA derived lipid mediators (figure 2). Differences between disease groups were, however, only confined and specific for the CSF. Thus, no changes were observed in the serum compartment. In the CSF, no differences between disease groups were observed in substrate levels (precursor lipids) of AA (*P = *0.69) or docosahexaenoic acid (DHA, *P = *0.69). Here, the CSF levels of AA and DHA correlated closely among all patients (*R^2^* = 0.9). In the active MS group the intermediate, AA-derived molecule 15-hydroxyeicosatetraenoic acid (15-HETE) reached significantly higher levels (4.4 pg/ml ±0.8 [mean ± SEM] vs. 2.2 pg/ml ±0.63, *P<*0.05), and this was also the case with the pleiotropic inflammatory mediator PGE_2_ (1.27 pg/ml ±0.27 vs. 0.65 pg/ml ±0.12, *P<*0.01), Thromboxanes, leukotrienes and prostaglandin D2 remained unchanged.

Among the anti-inflammatory and proresolutive lipid mediators, lipoxin A_4_ (LXA_4_) was not significantly increased in patients with active disease compared to those with inactive disease (figure 2B). As the 15-HETE level is enhanced (which serves as marker for the LXA_4_ pathway), we cannot exclude the possibility that LXA_4_ already metabolized within the snap shot time and that LXA_4_ levels were thus underestimated. In contrast, the DHA-derived resolvin D1 (RvD1) was increased in CSF of patients with highly active MS (0.89 pg/ml ±0.41 vs. 0.12 pg/ml ±0.05, *P = *0.014). Also, the DHA-derived neuroprotectin D1 (NPD1) reached detectable levels only in highly active MS patients. No correlation was found between mediators along the synthesis pathways (e.g. 17-HDHA and RvD1, *R^2^* = 0.025; 15-HETE and LXA_4_, *R^2^* = 0.094) suggesting a non-saturated relationship between substrate and effector molecules.

## Discussion

In this first comprehensive analysis of lipid mediator resolution agonists in human CSF we report that patients with ‘highly active’ and ‘less active’ MS not only had a different CSF expression of pro-inflammatory mediators, but also differed in their synthesis of proresolution lipid mediators. We were able to corroborate the elevated PGE_2_ and 15-HETE CSF levels that were recently reported in MS patients as a quantitative CSF marker of neuroinflammation and oxidative stress. [6] Importantly, no difference was observed in serum levels of PGE_2_ and 15-HETE, suggesting CNS-specific effects. This finding supports the well-established concept of compartmentalized effects in MS, as seen for several biomarkers [15].Interestingly, we detected a relationship between disease severity and the proresolution mediator RvD1, which has been shown to diminish inflammation caused by oxidative stress [16] and to dampen leukocyte trafficking into and clear macrophages out of inflammation sites. [8] Given a dichotomous resolution phenotype in humans controlled by proresolution mediators categorized in ‘early resolvers’ (low initial but persistently increasing) and ‘delayed resolvers’ (high initial but short-lived peak of resolution agonists insufficient to prevent chronic inflammatory disease), the ‘highly active’ cohort might qualify for the ‘delayed resolver’ group, since all CSF samples were derived during or shortly after the inflammatory relapse. [17] Another biological explanation would be that in the ‘highly active’ MS cohort increased CSF cell numbers would represent a higher synthesis capacity of the lipid mediator resolution agonists. In contrast to RvD1, the AA-derived LXA_4_ was not significantly upregulated in the CSF of ‘highly active’ MS patients, suggesting a differentially regulated resolution program defining a ω-3 PUFA versus a ω-6 PUFA hierarchy in response to an inflammatory stimulus within a spatio-temporal resolutive context. Furthermore, since endogenous LXA_4_ synthesis is limited despite sufficient substrate (15-HETE) which is in line with a defective “lipid mediator class switch” [18], it might be worthwhile analyzing whether aspirin-triggered epimeric LXA_4_ levels can also be boosted in the CSF of MS patients, as has been demonstrated in the serum of healthy subjects taking low-dose aspirin. [7] Disruption of 12/15-lipoxygenase (which produces LXA_4_) results in a break of immunological self-tolerance [19], which is impaired in MS. Thus, increased levels of aspirin-triggered epimeric LXA_4_ might support anti-inflammatory/pro-resolutive pathways and thereby be more specific than applying additional substrate by ω-3 & ω-6 PUFA supplemented nutrition.

The measurements reflect the lipid mediator profile at the time of clinical examination. Thus, although using several control metabolites, the technical approach cannot exclude that decreased levels of lipid mediators result from an increased rate of lipid metabolism/inactivation rather than decreased formation. Sample collection was optimized for analysis of frozen material which has repeatedly been shown by us and many other groups to result in robust disease- or inflammation-specific lipid mediator profiles not seen in healthy controls. However, we cannot rule out that class-specific degradation may affect the measured levels of selected analytes in both patient groups. Limited amounts of patient material did not allow for exclusion of any storage-related degradation, as human specimens would have to be spiked with specific labeled analytes.

The clinical outcome of MS patients might not be determined solely by the levels of pro-inflammatory mediators, which have conceptually dominated diagnostic and interventional approaches in past, but also by a milieu of resolution agonists at the lesion site. Further prospective longitudinal studies are needed to clarify whether clinical disease progression as measured with the Expanded Disability Status Scale (EDSS) correlates with the presence and levels of resolution agonists, thereby influencing disease longevity and eventually its termination.

## Supporting Information

Table S1 MRM parameters and analytes
(DOC)
