# Supplementary material for: Proresolution Lipid Mediators in Multiple Sclerosis — Differential, Disease Severity-Dependent Synthesis — A Clinical Pilot Trial
Source: PLoS One. 2013 Feb 8;8(2):e55859. doi: 10.1371/journal.pone.0055859 (PMC3568070; doi:10.1371/journal.pone.0055859)
Supplement: Table S1 — MRM parameters and analytes (DOC) [file pone.0055859.s001.doc]

**Supplementary Table S1**

MRM parameters and analytes

| **Analyte** | **Parent Ion** | **Transition Ion** |
| --- | --- | --- |
| **DHA Metabolome** | **Q1** | **Q3** |
| DHA | 327 | 283 |
| 4-HDHA | 343 | 101 |
| 7-HDHA | 343 | 141 |
| 14-HDHA | 343 | 205 |
| 17-HDHA | 343 | 245 |
| RvD1 | 375 | 141 |
|  | 375 | 215 |
| RvD2 | 375 | 175 |
|  | 375 | 203 |
| Maresin 1 | 359 | 250 |
| NPD1 | 359 | 153 |
| 22-OH-NPD1 | 375 | 271 |
| **AA Metabolome** |  |  |
| AA | 303 | 259 |
| 5-HETE | 319 | 115 |
| 12-HETE | 319 | 179 |
| 15-HETE | 319 | 175 |
| LXA_4_ | 351 | 115 |
| LXB_4_ | 351 | 163 |
| LTB_4_ | 335 | 195 |
| 20-hydroxy-LTB_4_ | 351 | 159 |
| 6t-LTB_4_, 6t-12epi-LTB_4_ | 335 | 183 |
| LTC_4_ | 624 | 272 |
| LTD_4_ | 496 | 177 |
| LTE_4_ | 438 | 289 |
| TXB_2_ | 369 | 169 |
| PGE_2_ | 351 | 271 |
| PGD_2_ | 351 | 189 |
| PGF_2α_ | 353 | 193 |
| 6-keto-PGF_1α_ | 369 | 245 |
| **EPA Metabolome** |  |  |
| EPA | 301 | 257 |
| RvE1 | 349 | 195 |
| PGE3 | 349 | 269 |
| 12-HEPE | 317 | 179 |
| 15-HEPE | 317 | 255 |
